# The E Sibling Project – exploratory randomised controlled trial of an online multi-component psychoeducational intervention for siblings of individuals with first episode psychosis

**DOI:** 10.1186/1471-244X-13-123

**Published:** 2013-04-26

**Authors:** Jacqueline Sin, Claire Henderson, Vanessa Pinfold, Ian Norman

**Affiliations:** 1 Florence Nightingale School of Nursing & Midwifery, King's College London, 57 Waterloo Road, London, SE1 8WA, England; 2 Institute of Psychiatry, King's College London, 16 De Crespigny Park, London, SE5 8AF, England; 3 The McPin Foundation, 32-36 Loman Street, London, SE1 0EH, England

**Keywords:** Siblings, Brothers, Sisters, First-episode early onset psychosis, Family, Randomised controlled trial, Psychoeducation, Peer support, Health education

## Abstract

**Background:**

Siblings of individuals with first episode psychosis are natural partners to promote service users’ recovery and are themselves vulnerable to mental ill health due to the negative impact of psychosis within the family. This study aims to develop and undertake a preliminary evaluation of the efficacy of an online multi-component psychoeducational intervention for siblings of individuals with first episode psychosis. The impetus for the intervention arose from siblings' expressed needs for peer support and information on psychosis, coping and management strategies for common symptoms and ways to promote recovery.

**Methods/Design:**

The project design draws on the Medical Research Council framework for the design and evaluation of complex interventions. Mixed methods comprising collection of qualitative focus group data, systematic review and expert advisory group consultation are used to develop the theoretical basis for and design of the intervention. This protocol focuses on the modelling and piloting phase which uses a randomised controlled trial with factorial design to test the efficacy of the intervention. Outcome data on participants’ mental wellbeing, knowledge, perceived self-efficacy and experiences of caregiving will be assessed at baseline, at end of the intervention (10 weeks later) and at 10 week follow-up. In addition, a post-intervention semi-structured interview with 20% of the participants will explore their experiences and acceptability of the intervention.

**Discussion:**

This multi-component online psychoeducational intervention aims to enhance siblings' knowledge about psychosis and their coping capacity, thus potentially improving their own mental wellbeing and promoting their contribution to service users’ recovery. The factorial design randomised controlled trial with a supplementary process evaluation using semi-structured interviews and usage-monitoring will collect preliminary evidence of efficacy, feasibility and acceptability, as well as feedback about the barriers and strategies to using such an innovative resource. The RCT will provide data for estimating the likely effect size of the intervention on outcomes for siblings and inform the development of a definitive future trial.

**Trial registration:**

Trial registration: ISRCTN01416694

## Background

Over 80% of the general population in the U.K. has at least one sibling, and the sibling relationship often outlives any other kind of relationship, including marriages and parenthood [1]. Siblings share *‘a common social, cultural and genetic heritage that often reaches into old age’*[2]. As such, the sibling relationship can be particularly influenced or influential when one sibling develops a severe mental illness. It has been argued that the quality of the sibling relationship, especially during adolescence and early adulthood, is a predictive factor in the siblings’ future involvement in caring for their brother or sister with severe mental illness [3], as well as being associated with a higher quality of life [4] and a more promising recovery trajectory [5,6] in individuals with a diagnosis of psychosis. However, despite the significance of the sibling relationship and policy guidelines advocating a family inclusive approach across mental health services, especially Early Intervention in Psychosis Services (EIPS) [7], front-line service provision falls short in this respect. There remains a paucity of research into effective interventions for siblings of individuals with first episode psychosis (FEP) [8-10].

Psychoeducation, information-giving on the condition and its management [7,11], is among the most effective of the evidence-based psychological interventions that has long been implemented as both an individual and a group approach to service users of severe mental illness and their family members or carers (see [11] for an updated Cochrane Review). It is frequently hypothesised that the effectiveness of psychoeducation can be explained by its impact on knowledge, on stress appraisal and on coping, and subsequently perceived subjective burden and self-efficacy (for example: [12,13]); so building upon the theory of stress and coping first coined by Lazarus in the 1960s [14]. Meanwhile, a recent qualitative research study of FEP siblings’ needs and experiences [9,15] highlighted siblings’ needs for robust, dynamic and accessible psychoeducational information on psychosis, coupled with peer support, practical coping and management strategies for common psychotic signs and symptoms, adjustment to loss and changes, and ways to promote recovery in their ill brother or sister. Most siblings reported experiencing a range of emotions, for example: worry, guilt, loneliness and stigma, that affect their own wellbeing, whilst some also reported developing resilience within themselves and their families [6,9].

Traditional and conventional psychoeducation delivery through face-to-face sessions with mental health professionals or accessed through statutory mental health services has failed to reach siblings successfully hitherto [9,15,16]. A high proportion of siblings of individuals diagnosed with FEP are in full-time education or are of working age and many have busy lives. Thus any service development targeting siblings demands flexible, dynamic and innovative measures using modern information and communication technologies [17]. There is a handful of research studies showing promising benefits for multi-component online psychoeducational interventions, covering a range of long-term and severe diseases including dementia, diabetes, and cancer. Multi-component interventions for these conditions commonly include: information sharing, networked peer support, electronic resources and facilitated discussion forums [18]; but much less work has been done in the field of severe mental illness [19,20]. To date, there are only three known exploratory controlled trials evaluating online psychoeducational interventions with service users with psychosis and their family carers in the U.S.A. [19,21,22] and with service users with bipolar disorder in Australia [23]. In the UK, a few online blogs and information-giving websites have been established by leading charities over the last few years (for example: http://www.sibs.org.uk[24] and http://www.rethink.org/siblings[25]). However, there is no known evaluative study of any such intervention for people with severe mental illness and their families, let alone siblings.

### Justification and rationale of the current project

The need for a robust, flexible and effective psychoeducational intervention that also provides an element of peer support for siblings especially when they are in their late teenage and early adulthood is clear [9,16]. There have been a number of successful and effective online psychoeducational interventions for service users and their family members across a wide range of long-term severe illness, but such interventions are under-developed in the mental health field. A few charities in the U.K., Australia, Canada and the U.S.A., prompted by siblings’ own initiatives and demands, have been running information-giving and network support web-sites for siblings over the last few years. However, there are no known evaluations of the impact of these initiatives. In a time of evolving technological advances and ever-increasing emphasis on cost-effective and evidence-based interventions promoting self-management, development of an online psychoeducational intervention incorporating various components and resources indicated for this population together with a rigorous evaluation, is timely.

### Aims of the project

This paper describes the rationale and protocol for a factorial designed randomised controlled trial targeting siblings of young people diagnosed with FEP who are receiving support from local EIPS. The aims of the overall “E Sibling Project” are to:

I. Develop a multi-component resource combining health information and peer support enlisting research evidence and siblings’ views.

II. Optimise the IT and e-learning technologies in the design and delivery of the intervention.

III. Undertake a preliminary evaluation of the efficacy of the intervention.

The trial has the following objectives:

IV. To optimise the intervention

V. To determine trial parameters

VI. To provide preliminary evidence of the efficacy of the intervention in terms of impact on siblings’ mental wellbeing; knowledge; perceived self-efficacy in coping and experiences in caregiving.

VII. To optimise the design of a future full scale trial

## Methods

**Figure 1:**
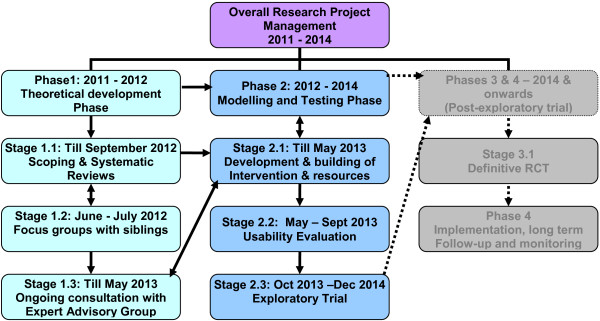
Design of the study using MRC complex intervention framework, inclusive of time schedule for various phases.

The design of this study draws upon the UK Medical Research Council’s complex interventions framework [26] to address the key elements of the development and evaluation of an online multi-component psychoeducational intervention for siblings of individuals diagnosed with FEP. The intervention is “complex” according to the MRC definition [26] because it comprises a number of and interactions between components within the intervention, that subsequently impact on a number of variable outcomes. The components are: psychoeducation and peer support, each of which is a complex intervention in its own right, and each may work independently as well as inter-dependently in exerting their effects. The potential outcomes include: siblings’ mental wellbeing, knowledge, self-efficacy and coping. Another issue for consideration in complex interventions is the number and difficulty of performing behaviours required by those delivering and/or receiving the intervention, such as those involved in this intervention by the sibling-participants. These complexities demand a factorial designed RCT in which not only primary outcomes and multiple secondary outcomes are investigated, but also the process evaluation measures to understand participants’ experiences and actual usage of the intervention. The project comprises a number of phases using mixed research methods, as outlined in Figure 1. The study was reviewed and approved by UK NHS Research Ethics Committee process (REC approval reference number: 12/LO/1537).

**Figure 2:**
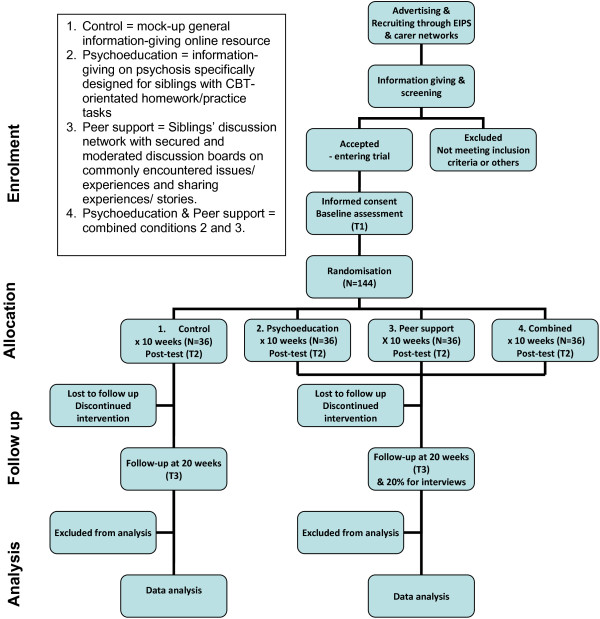
Progression of participants through the phases of the trial.

The RCT comprises an exploratory trial, to complete the modelling and piloting phase of the MRC 4-phased framework [26]. The RCT employs a 2 by 2 factorial design, as illustrated by Figure 2. The factorial design gives the RCT 4 arms: 3 treatment conditions (covering: psychoeducation alone; peer support alone; and combined psychoeducation and peer support) and a control condition. This design is chosen to fit with the two active components in the intervention, i.e. psychoeducation and peer support. Considering that these two ingredients may work independently as well as inter-dependently, the three treatment arms allow an investigation not only to determine the effectiveness of the combined psychoeducation and peer-support treatment condition, but also of how each ingredient impacts independently on particular participants’ outcomes, minimising any contamination [26,27]. Hence, the factorial design RCT will aid our theoretical understanding of the intervention, how it works, how its ingredients interact with each other and ultimately how it supports changes in the targeted outcomes. Furthermore, the results using the factorial design will inform the optimal intervention to be used in the full scale trial.

### Design

#### Randomisation, treatment allocation and blinding

This is a factorial design RCT in which participants are randomised and allocated to 4 conditions: 3 treatment conditions in addition to treatment as usual (control). The three treatment arms will allocate participants into either: psychoeducation alone; peer support alone; or combined psychoeducation and peer support. Screening for eligible participants against inclusion and exclusion criteria is conducted online through screening questionnaires. All eligible participants will then be allocated to one of the three treatment or the control condition. Randomisation is done using computer-generated permuted blocks and is carried out by an independent Clinical Trial Unit (CTU) to ensure blindness. Eligible participants will receive a computer-generated account with username and a unique password as well as the treatment group they have been allocated to.

### Participants

#### Recruitment & Informed consent

Advertisement and recruitment will be conducted through a website developed and dedicated to delivery of the online information and peer support intervention produced by King’s College London; Early Intervention in Psychosis Services (EIPS); local carers groups and networks; and the Mental Health Research Networks (MHRN) across South-East England, including London. Care co-ordinators and clinicians working in EIPS in five NHS Trusts across South-East England will be given information about the trial in a series of presentation by the trial team. Clinicians will help identify potentially suitable participants from their case load of service users to the trial co-ordinator, with the service users’ and/or potential participants’ consent. The trial co-ordinator will make contact and provide further information about the trial to all potential participants. Individual consent from the participants will be sought following comprehensive information-giving and a consideration period of at least 3 days. Presentations will also be made at (family) carers and siblings events hosted by Rethink Mental Illness and other carers-related organisations/ networks over the period. Interested siblings may refer themselves directly to the research team to join the trial after the same information-giving, consideration and screening process.

Each of the five NHS Trusts covers a population of about one million and each has an EIPS caseload ranging from 400 to 600 service users at any one time. EIPS are dedicated mental health services for people aged between 16 to 35 who are affected by a first-episode psychosis [7]. The provision of service by EIPS usually starts from immediately following the onset of first episode psychosis, a critical period in determining long-time trajectory in recovery [5,7], and the treatment period is usually about three years. Following that, EIPS usually transfer the care of service users back to their GPs or to secondary mental health services for long-term support, e.g. community mental health teams. The time required to recruit 144 participants is estimated as 20 weeks. Contingency plans to address any short-fall in the recruitment include recruitment of additional participants to compensate for anticipated 20% attrition, extra time for recruitment and extension of the recruitment area to additional NHS Trusts within the geographical regions.

#### Inclusion criteria

This trial targets siblings of individuals diagnosed with FEP directly. Hence, 144 siblings will be recruited who fulfill the following inclusion criteria:

&z.squf; aged 16 or above;

&z.squf; has a brother or sister receiving service from a local EIPS at the time of joining the trial;

&z.squf; is based within the Greater London or Berkshire area themselves;

&z.squf; has had contact at least weekly with their ill brother or sister on average over the previous 3 months.

&z.squf; can read and understand English language in usual online communications.

&z.squf; has daily access to internet use.

#### Exclusion criteria

Regrettably, we cannot accept siblings with the following criteria into the trial:

&z.squf; individuals unable to give informed consent;

&z.squf; those affected by a mental illness themselves that requires treatment and care from a secondary/ specialist mental health services (e.g. seeing a psychiatrist at out-patient clinic regularly; under the care of a community mental health team; has a care co-ordinator).

Since the age limit for sibling-participants is aged 16 or above, all participants will be able to consider their own rights and interests and give informed consent without parental guidance or assent. The peak age of first psychotic onset is estimated as late teenage and early adulthood, around 22 years of age in the UK [7]; it is estimated that majority of the siblings for people with FEP who have had direct experience of supporting their brother/sister will have an age range of teenage to young adulthood.

Considering the intervention is an online resource with a mixture of textual, audio and visual information and the trial is a feasibility pilot, the participants are required to have a basic level of ICT and English which are required to make use of the intervention. Nonetheless, IT and technological support will be available through emails and phone-contacts between the participants and an IT support service located in parallel to the research team.

#### Sample size

The exploratory trial is a feasibility pilot study to establish a preliminary estimate of the efficacy of an innovative intervention. The trial is not designed for hypothesis testing [24,25]. The pragmatic sample size of 120 participants for the trial was selected with reference to the general rule of thumb recommended by Lancaster et al. [27], taking account of the factorial design of this exploratory trial and the primary and secondary outcome measures to be investigated. A sample of 30 per arm is considered sufficient to obtain estimates of the values of outcomes for each arm and thus an estimate of the effect size which would then be used to inform the design of a definitive trial. To ensure the rule of thumb minimum sample of 30 will be retained in each arm, additional participants (i.e. N=144, n=36 in each arm) will be recruited to allow for an estimated attrition rate of up to 20%.

#### Outcome data collection

Assessment of participants is conducted through completion of online questionnaires for demographic data and outcome measures at baseline (T1=week 0); at end of intervention (T2=week 10) and at 10-week follow-up (T3=week 20). Such data is collected directly from the participants via online survey tool inbuilt within the trial.

#### Outcome measures

Sibling-participants will be assessed on the primary outcome and three further secondary outcomes at three time-points. Data collection using the following outcome measures tools is through online completion by all participants across the treatment and control conditions.

The primary outcome of this intervention is siblings’ mental wellbeing measured by a self-administered tool, the Warwick-Edinburgh Mental Wellbeing Scale (WEMWBS) [28]. WEMWBS has been validated to assess the impact of interventions on the mental wellbeing of a population sample [28]. Its brevity of 14 positively-worded items and 5 response categories make it ideal for use in this trial.

There are three secondary outcome measures, including: knowledge of mental health using Mental Health Knowledge Schedule (MAKS) [29]; self efficacy using the Assessment of Perceived General Self-Efficacy (APGSE) [30], and negative and positive experience of caring using Experience of Caregiving Index (ECI) [31].

The intervention, especially its psychoeducation component, is expected to impact on participants’ knowledge about psychosis. The MAKS is an ideal tool to assess and track stigma-related mental health knowledge amongst the sibling-participants. It has been validated for use in both face to face interviews and for online data collection [29]. To assess participants’ perceived self-efficacy with regard to optimistic beliefs about coping with a large variety of stressors, regardless of their circumstances, the online version of the APGSE [30] is used. This version, with a 10-item scale, has been found to be valid and feasible for online use. Finally, for assessing the sibling-participants’ experiences of caregiving, the ECI [31] will gather relevant information from siblings. The ECI contains 66 items covering 10 sub-scales of both positive and negative experiences of caregiving. In this trial, all 10 sub-scales inclusive of further break-down questions will be presented in a format of brief statements to the participants via an online questionnaire. The 10 sub-scales are: effects on family; stigma; negative symptoms; difficult behaviour; dependency; loss; positive personal experiences; and good aspects of the relationship with the service users; problems with services; and need for back up.

#### Process evaluation

Data on patterns of uptake and usage (number of hits, time spent on-line etc.) from all participants will be monitored to correlate efficacy with usage and to ascertain feasibility.

After completion of the follow-up questionnaire data (T3=week 20), about 20% of the participants (n=20) across the three treatment groups will be purposively selected for interview to explore their experiences of using the resource and the perceived acceptability of the intervention. Purposive selection is used to identify the participants to ensure representation of diverse experiences and views from those with ethnic minority backgrounds, frequent and infrequent users whose experiences may impact on their usage and outcomes. Conventional delivery of psychosocial interventions (i.e. face to face) is known to be less likely to be offered to or accessed by people with Black and Minority Ethnic backgrounds (BME) [7]. The purposive sampling for the process evaluation aims to investigate if the online delivery and medium addresses this well-known access and equality issue, in addition to seek to understand their experience and perceived acceptability of the intervention. A post-intervention individual semi-structured interview will be conducted in person, via phone or online conference, depending on the participants’ preference. In addition, data on patterns of uptake and usage from all participants, e.g. number of hits and time spent on the treatment condition allocated will be monitored to correlate effectiveness with usage and to ascertain feasibility.

### The intervention

#### Development and building the intervention

The development of the online multi-component psychoeducational intervention is informed by the previous stages of the overall project, i.e. the theoretical development phase in the first 18 months. The theoretical phase of the overall study comprises two stages, including a mixed-method systematic review and a focus group study with siblings (See Figure 1).

The systematic review conducted in Phase I, and recently completed, has identified the evidence base of psychoeducational interventions for families and relatives of people with psychosis and the common essential ingredients, implementation factors (both facilitators and barriers) in those effective interventions [32]. The review, also informs the theoretical modelling of the related concepts and essential ingredients of the intervention to better understand how each ingredient works independently as well as interdependently to produce the desired primary and secondary outcomes in the target population. We have also reviewed outcome measurement tools used by previous studies and modified them as needed to suit the sibling-participants and the online medium of data-collection.

The theoretical understanding accomplished was further built on in a participative exploratory study involving siblings of individuals affected by psychosis. Focus group methodology was chosen because we were interested in understanding a range of perspectives and valued the insights gained through group discussion between siblings [33]. The views of siblings were as crucial as the evidence base drawn from the systematic review to ensure that the intervention addresses siblings’ specific needs in a contemporary context [34]. During the focus group discussions involving 14 siblings conducted in mid 2012, they were asked to help design the intervention in a way that would optimise utilisation, access and relevance of the intervention to their own needs and life-styles. They were also asked about what services they would like and in what medium and content they would like these services to be covered. Potential barriers were identified and strategies to overcome them developed [35].

The intervention is currently being built and engineered through intensive input of an Expert Advisory Group (EAG) and e-learning expertise, across the theoretical development and modelling phases in the MRC framework (see Figure 1). The EAG will comprise siblings (50%), service users, parents, mental health clinicians, youth organisation/ charity personnel and e-learning IT experts. Guided by the conceptual framework and design-content produced through the previous stages discussed above, the EAG will advise on the design and content of the psychoeducational intervention resource package built through a process of consultation forums, including: design and selection of the scripts / content for the information section, provision of textual/ audio-visual inputs and blog-building for the peer support section. It will also validate and review the online intervention being built, along the process. The process of these 2 parallel stages will follow the development cycle published recently by National Institute for Health Research Service Delivery and Organisation (NIHR) programme [36], reporting their successful experience of developing an online training resource. There will be five build steps in the development cycle: 1. draft architecture and content; 2. mock up of shell; 3. fill shell with material; 4.cross inference and integrate materials, and 5. prepare for piloting.

Finally, a usability evaluation (see Figure 1) will test the usability of the online intervention with 20 siblings, prior to the trial. The usability test will adapt Poulson et al’s framework [37] to collate data on ease of use, accessibility, logistic of navigation, and trial use of pre- and post outcome measures within the intervention. Both objective observation and subjective feedback from the usability evaluation users will be analysed and used to refine the intervention, ready for the exploratory trial.

#### Design and content of the intervention

It is anticipated that the final intervention will comprise multiple components and the following characteristics: psychoeducation focusing on information-giving on psychosis, common treatment and management strategies for symptoms; and a peer support element that uses a virtual discussion network with secured and moderated discussion boards on commonly encountered issues and experiences to facilitate mutual sharing and discussion between siblings. There are also supplementary links to relevant resources inbuilt within the psychoeducation and peer support components.

**Figure 3:**
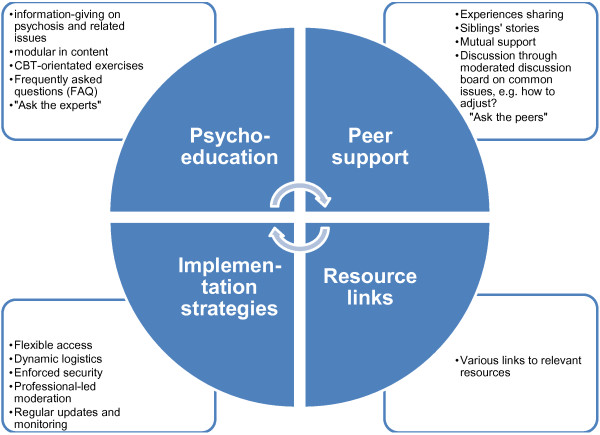
Conceptual framework of the online intervention for siblings.

Within the two major components (i.e. psychoeducation and peer support) a modular approach will be used to incorporate both information-giving and active strategies that link learning/ knowledge to everyday life practice, as informed by the identified essential ingredients of effective interventions [32,38]. Most of these strategies could be described as cognitive-behavioural orientated and involve online and off-line practice/ homework tasks. The modular information-giving covers topics identified by siblings themselves in addition to the evidence-base as identified by the systematic review. These include, for example: causes of psychosis; common treatments; how could I help as a brother or sister?; looking after myself; what does the future hold? In addition to the textual medium as a major way of information giving, audio and video links will be used. Online discussion forums resembling a virtual support group for siblings, where they can discuss shared issues and exchange views with one another, is another important feature of the intervention. Considerations of confidentiality and anonymity, group safety and cohesiveness, are addressed in the design and delivery of the intervention, with additional IT-related issues. For instance, the online peer support is a closed virtual group limited to participants, all participants will use a username on the online discussion forum without any further information linked to themselves or their families. There will be professional-led facilitation/ moderation and regular monitoring across the online resource. A conceptual framework of the intervention design is illustrated in Figure 3.

The intervention is designed for siblings’ use at their own home or base. They can access and use the intervention 24/7. Through online IT or phone helpdesk, support is provided depending on the participants’ preferences. For instance, any participants who experience problems with their username or forget their password, can email for a reminder or reset their password. An online tutorial is also available to demonstrate the features and functions of the intervention.

#### Control condition

The control condition is a mock-up focusing on severe mental illness accessed through the same trial website. It consists factsheets for siblings of people with mental illness which represent the best information available to them currently.

#### Intervention exposure

All participants across the four conditions will be asked to use the resources over the duration of the trial, i.e. over 10 weeks with a recommended usage of 1 to 2 hours per week. Weekly reminder via emails will be generated and sent to all participants to reinforce their use of the intervention. An online support centre is available to participants for queries over their usernames, passwords and/or any problems with access.

### Analysis

#### Quantitative data

Ordinal outcome data collected at baseline (T1), post-intervention (T2) and 10 week-follow up (T3) will be transferred into ranked scores, ready for analysis. Descriptive statistics focusing on variance and confidence interval estimation will then be performed. A variety of tests will be employed to compare within and between subjects, for example: Mann–Whitney and Wilcoxon Tests. Also ANOVA will be performed on ranked data. Following the framework and its elements for optimising trial parameters [27], the trial analysis will also provide data for calculating sample size and the effect size estimate for a future definitive trial. All analyses will be carried out using SPSS (SPSS, Inc.; Chicago, Illinois).

#### Qualitative data

The qualitative data on participants’ experiences and perceived acceptability of the intervention will be analysed using Ritchie et al’s thematic analysis framework [39], assisted by NVivo (http://www.qsrinternational.com). This method of analysis is chosen because it is suited to the analysis of large qualitative data sets and enable feedback to the participants. Thematic analysis using Ritchie et al’s framework [39] will classify and organise data according to key themes and concepts. Three broad stages are involved in the analytical process. First, initial themes are identified by “indexing” transcript. These themes will then guide formation of a framework within which transcribed material is summarised. Key categories are then identified to help described the data. Finally, patterns of association are explored and explained. To ensure our analysis is grounded in the data, it will be performed in parallel to the qualitative data collection, so the developing themes and framework of analysis can be tested and validated in latter data [33,39]. The data-analysis will be complete when “saturation” of themes is reached. We estimate that about 15 interviews will be needed though the guiding principle of saturation will take precedence in determining the completeness of the qualitative analysis [33,39].

## Discussion

The design of the intervention is based upon the stress-appraisal and coping theory [14] that is commonly used in conventional psychoeducational interventions targeting family members and relatives (for example: [12,13]). The needs for information and peer support have been identified by siblings themselves as well as in a prior qualitative study targeting the same population group [9,15]. As such, some conventional outcome measures like the ECI [31] and the Assessment of Perceived General Self-efficacy [30], together with more recent development, like the MAKS [29] and WEMWBS [28], are used in this trial. Whilst retaining some frequently used outcome measures targeting changes in knowledge and self-efficacy, we have decided against including some other traditional outcome measures that have been used historically to determine changes in family members’ perceived burden and expressed emotion [12,13]. Our earlier research on siblings’ experiences and needs of service identified that most of them do not regard themselves as (primary) “carers” [9,15]. Instead, most siblings see their parents shouldering the primary carer role and their own contribution being supporting their ill brother or sister as well as their parents.

Considering the dearth of research with siblings of people with FEP, this trial serves as an exploratory study which has, as one of its aims, to produce an estimate of the value of outcomes. On a more general level, the trial will test out the feasibility and efficacy of an innovative intervention delivered online, which draws its theoretical base from well researched psychoeducation and peer support which is conventionally delivered face-to-face. Considered together with data on usage patterns (e.g. number of hits, frequency and duration of use) which is inbuilt within the online medium and is collected as a matter of routine, further analysis could be conducted on feasibility and correlation between efficacy and usage.

The online delivery process of the intervention arguable provides further benefits in enhancing the qualities of the randomisation, concealment (to participants) and double blinding (to both the participants and the researchers) as all procedures are pre-set by the CTU and IT technicians, independent of the research team who is responsible for data analysis. Data collection is direct from the participants to the online outcome measure tools, to minimise any possible contamination brought about by a researcher interviewing the participants.

The intervention developed through the E-Sibling Project has the potential to be disseminated widely at minimal cost given its online design and delivery medium, in the medium term future. The intervention will provide a service to siblings who are currently often invisible to the statutory service and voluntary organisations providing a service for carers.

## Competing interests

All authors declared that they have no competing interests.

The research team expects to automatically own the copyright of the online psychoeducational intervention which will be produced and evaluated as an end-product of this project. We also expect that the development of the online psychoeducation intervention through this research project may lead to the production of Intellectual Property Rights (IPR) on design and technology in the specialty of e-learning technology. Nonetheless, it is our intention that the end product will be made freely accessible on a not-for-profit basis following completion of the project.

## Authors’ contribution

JS designed the study with advice and guidance and coordinates the project overall. She is also the holder of the NIHR Doctoral Research Fellowship grant. JS, IJN and CH drafted this paper. IJN and CH provide research supervision to JS. VP gave consultation and advice on the design of the study, represents the McPin Foundation in the collaboration and was formerly the study link to Rethink Mental Illness. IJN, CH and VP made a substantial contribution to the design and management of the project. All authors have contributed to the writing and have read and approved the final paper.

## Pre-publication history

The pre-publication history for this paper can be accessed here:

http://www.biomedcentral.com/1471-244X/13/123/prepub
